# Notes on Current Topics

**Published:** 1909-01-30

**Authors:** 


### Motoring Notes.

### NOTES ON CURRENT TOPICS.

### A Defence of Single Cylinders.

I have lately had the opportunity of reading an article on motors for medical men in a medical journal published in the Midlands, in which the author, although acknowledging that the single-cylinder car is undoubtedly the simplest to keep in order and most economical in upkeep, asserts that it is difficult to start, noisy and shaky when standing, and slow at hill climbing. He further states that the two-cylinder car is little better in the way of vibration and easy starting, and always requires considerable winding. He concludes by saying that the medical man who wishes to work his practice with a motor-car and at the same time derive pleasure from the pastime and freedom from difficulties, would be well advised to buy a four-seated car with a four-cylinder engine of not less than 10 horse-power, fitted with accumulators and coil of the best quality obtainable; and adds that such a car can be bought from makers of established reputation for about £250.

I have no hesitation in saying that these statements are totally at variance with my own experience of motors spread over many years. Taking the last point first, I should like to know the names of really high-grade four-cylinder, four-seated cars that can be bought for £250. I do not believe a really high-class (and none other is suitable for a medical man's use) four-cylinder car can be purchased for anything approaching that figure.

I have at present on order a single-cylinder, two-seated car of 6.2 horse-power, R.A.C. rating, which, without extras, is costing me £276. This car is, however, of the very highest grade, and will give me far less trouble, be quite as smooth in running and infinitely cheaper to maintain than any low-priced four-cylinder car: and, I will venture to add, will not depreciate in value to anything approaching the same extent. It must not be assumed that I in any way personally object to a four-cylinder. Could I afford the initial cost and general upkeep of a four-cylinder car of the same class as the single-cylinder alluded to I should much prefer it, but the fact remains that I cannot, nor, I think, can the great bulk of medical men. A single-cylinder car of really high grade is infinitely superior in every way to a multi-cylinder at about the same price; for the latter must be inferior in design, workmanship, and material.

In regard to the statement that the single-cylinder is difficult to start, noisy, shaky, and a poor hill-climber, I can only conclude that the author of the article has no experience of a modern high-grade single-cylinder car, and that his experience of this type must have been confined to obsolete models. I have a small single-cylinder car now some six years old that always starts on the second turn of the handle, if not on the first. I can show him single-cylinder cars that are as quiet as many multi-cylinders. As to hill-climbing, I presume he has never heard of Mr. Douglas Fawcett's wonderful feats of Alpine climbing on an 8 horse-power car. Statements such as these about cars suitable for the medical man should not go unchallenged; and it is to be hoped readers of this journal may therefore avoid the very grave error of purchasing cheap multi-cylinder cars which may run in a satisfactory manner for the first year, but which after this period will prove a source of trouble and expense.

### Hoods for Doctors' Cars.

The subject of hoods for the doctor's car is one that is of importance, especially at the present season. Doubtless a well-made leather hood that comes well forward over the screen is the best protection against bad weather. This variety has, however, the drawback of being expensive, somewhat heavy, not easily removable, and of not being such an efficient dust screen in dry weather as the Cape cart type. The latter is light in weight, easily fixed on or removed from the car, in wet weather will turn a lot of rain, and in dry makes an excellent dust screen. Its disadvantages are that the cotton or other fibre cloths with which this type of hood is generally covered rapidly fade and become shabby, causing trouble and annoyance in addition to the expense of re-covering.

Recently, however, the Kamac Manufacturing Company, of Cross Lane Mills, Bradford, have placed on the market a material called Kamac for Cape hood covering, which they claim is rot-proof, does not stain or fade, and is much more durable than the ordinary cloth hitherto used for hoods. Kamac is a wool cloth made up from a natural coloured yarn, and so woven that there is a layer of rubber proofing between. In colour it is a light brown, and is distinctly handsome in appearance. It is made in two weights, the heavier having a lining of the same material as the outer side. The material is more expensive than cotton fabric, and for this reason many car manufacturers and body builders do not care to recommend it. I had a Cape hood covered with Kamac last June. When I ordered it my carriage builder told me that he had a material which always gave satisfaction and which he considered superior. I insisted despite this on Kamac, and my hood was covered with this material. As it happened a relative of mine had a hood made at the same time by the same firm, but of the material recommended. Before the end of the summer the covering of this hood had become piebald owing to the sun bleaching the folds when the hood was down, whilst mine never faded in the least degree. I also found that the hood could be folded wet and left so without the slightest rotting effect. In short I have found Kamac to bear out all the manufacturers claim for it, and I can most strongly recommend it as the very best covering for Cape hoods.

"Viator."

